# The Role of Ferritin in Health and Disease: Recent Advances and Understandings

**DOI:** 10.3390/metabo12070609

**Published:** 2022-06-30

**Authors:** Nikhil Kumar Kotla, Priyata Dutta, Sanjana Parimi, Nupur K. Das

**Affiliations:** Department of Molecular and Integrative Physiology, University of Michigan, Ann Arbor, MI 48109, USA; nkotla@med.umich.edu (N.K.K.); priyatadutta576@gmail.com (P.D.); saparimi@umich.edu (S.P.)

**Keywords:** ferritin, ferritinophagy, iron disorders, ferroptosis, COVID-19

## Abstract

Systemic iron homeostasis needs to be tightly controlled, as both deficiency and excess iron cause major global health concerns, such as iron deficiency anemia, hemochromatosis, etc. In mammals, sufficient dietary acquisition is critical for fulfilling the systemic iron requirement. New questions are emerging about whether and how cellular iron transport pathways integrate with the iron storage mechanism. Ferritin is the intracellular iron storage protein that stores surplus iron after all the cellular needs are fulfilled and releases it in the face of an acute demand. Currently, there is a surge in interest in ferritin research after the discovery of novel pathways like ferritinophagy and ferroptosis. This review emphasizes the most recent ferritin-related discoveries and their impact on systemic iron regulation.

## 1. Introduction

Iron is essential for almost all living organisms on earth. Its unique ability to donate and accept electrons makes it favorable for fundamental biological processes such as DNA synthesis and repair, oxygen transport, cellular respiration, heme synthesis, and immune function [1]. Although cellular iron deficiency is detrimental, iron excess is also potentially hazardous as its divalent form (Fe^2+^) generates toxic reactive oxygen species (ROS) via the Fenton reaction causing oxidative damage to the cells [2]. From the human health perspective, an iron imbalance is a major global concern, and both deficiency and overload disorders are prevalent in distinct geographical locations as well as in different genetic backgrounds [3]. In addition, patients with hereditary hemoglobinopathies, such as sickle cell disease and thalassemia, exhibit a complex form of iron disorders that represent both anemia and progressive tissue iron overload consequent to chronic hemolysis [4,5,6]. Mammalian iron metabolism is tightly regulated both at the cellular and systemic levels [7]. Although our body can extensively recycle its own iron, a regular supply of iron through diet (daily requirement is 10–20 mg, of which about 10% is absorbed) is critical for efficient homeostasis [1]. Dietary iron is absorbed by the duodenal enterocytes with the help of three main transporters, namely, the apical transporters divalent metal transporter 1 (DMT1), duodenal cytochrome b reductase (Dcytb), and the basolateral transporter ferroportin 1 (Fpn1) [8,9,10].

One of the most important components of cellular or organismal iron homeostasis is iron storage. Intracellular iron storage function is carried out by ferritin, which is structurally composed of 24 subunits of light (FTL) and heavy chains (FTH) that form a nano-cage complex to hold up to 4500 iron atoms [11]. Ferritin sequesters excess intracellular iron and stores it in a redox-inactive form for future use in conditions of deficiency or high demand. Cellular and systemic ferritin levels are not only crucial indicators of iron status but are also important markers of inflammatory [12], immunological [13], and malignant disorders [14,15]. Genetic alterations of ferritin are often associated with severe pathologies, and homozygous FTH deletion in mice is incompatible with life [16]. Conditional deletion of FTH in the intestine leads to iron overload in mice characterized by intestinal hyperabsorption and dysregulated systemic control by hepatic hepcidin highlighting its requirement for accurate intestinal iron absorption [17]. On the other hand, macrophage-specific FTH deletion in mice reduces intracellular iron levels and protects from high fat diet-induced metabolic disorders by lowering inflammatory and prooxidant damages [18]. In humans, a rare FTH mutation was identified in a Japanese family suffering from iron overload [19], and mutations in the FTL gene cause various ferritin disorders, e.g., hereditary hyperferritinemia with cataract syndrome, neuroferritinopathy, benign hyperferritinemia, etc. [20].

Intracellular iron homeostasis is mediated at both transcriptional and translational levels by iron regulatory proteins (IRPs) and iron-responsive elements (IREs) [21]. IRP has two subtypes, IRP1 and IRP2, both regulated by cellular iron and oxygen levels [21,22,23]. During low iron conditions, increased IRP levels lead to its enhanced interaction with the IREs present on either 5′-UTR or 3′-UTR of the mRNAs of iron-related genes (e.g., transferrin receptor 1(TfR1), DMT1, Fpn1, etc.). IRP-IRE interaction at 5′-UTR of both ferritin L and H mRNAs inhibits ferritin translation [24]. In addition to being a marker of iron status, ferritin is also one of the most important biological markers of inflammation. During inflammation, there is cytokine-mediated induction of ferritin H and L synthesis at both transcriptional and translational levels [25].

In recent years, we have gained more insight regarding ferritin function in the contexts of intracellular iron dynamics among different compartments, novel iron-dependent mechanisms related to cell growth and viability, and new diagnostic or prognostic indices related to infectious and/or inflammatory disorders, especially COVID-19. This review will discuss a few seminal discoveries that reshaped our understanding of ferritin’s role in biology.

## 2. Cytosolic Chaperones for Delivering Iron into Ferritin

**Figure 1:**
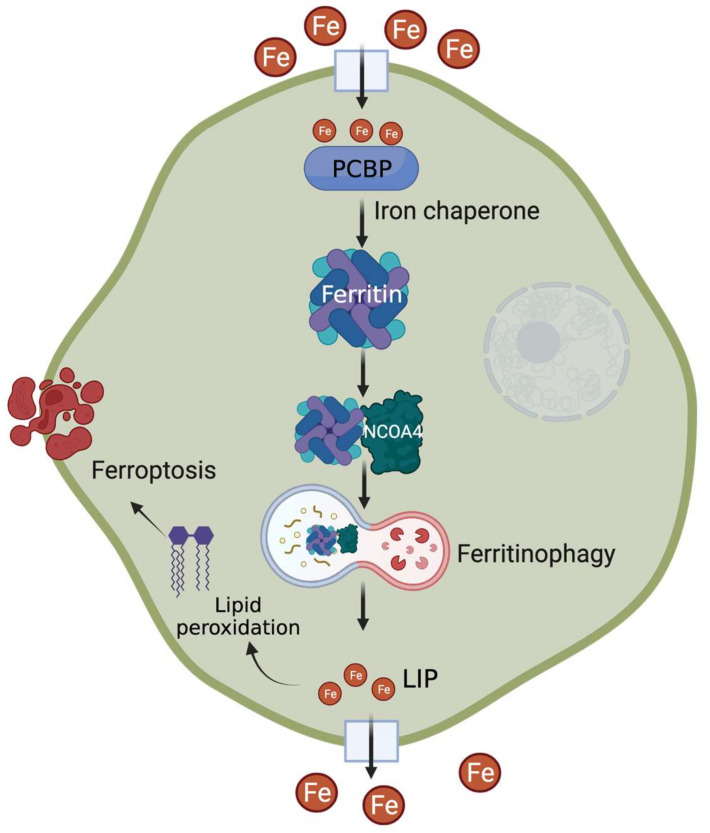
Recent advances in understanding the regulation of cellular iron storage mechanism. After cellular uptake, PCBPs act as iron chaperones to deliver iron to ferritin. During conditions of increased demand, NCOA4 acts as a cargo receptor for lysosomal degradation of ferritin, followed by increased labile iron pool (LIP) level. Excess iron contributes to lipid peroxidation-mediated ferroptosis.

The fate of intracellular iron is determined by the delicate balance between cellular iron usage and surplus. To ensure this, metabolically accessible iron, also known as cytosolic labile iron pool (LIP), must be efficiently distributed to its sites of utilization, transport, or storage. Although the mechanisms of ferritin regulation (e.g., translational regulation via iron response element (IRE)-iron regulatory protein (IRP) interaction at the 5′UTR) in response to cytosolic LIP status are well defined [24,26,27], the recent discovery of a cytosolic trafficking system that delivers iron to ferritin has significantly advanced our concepts of LIP dynamics. A member of the poly(rC)-binding protein (PCBP) family, initially known to be involved in the stabilization and translational regulation of RNAs containing C-rich motifs, PCBP1 was the first to be identified as a cytosolic iron chaperone that directly binds iron and loads it onto ferritin [28] (Figure 1). The ferritin-binding activity of PCBP1 is negatively regulated by cytosolic iron levels [29]. Subsequently, other PCBP members, namely PCPB2, PCPB3, and PCPB4, were also found to have iron chaperone activity with variable efficiency in iron delivery to ferritin [30]. Of note, PCBPs also play roles in the metallation of several iron-dependent enzymes: prolyl hydroxylases (PHDs), asparaginyl hydroxylase (FIH1), and deoxyhypusine hydroxylase (DOHH), highlighting their critical role in LIP distribution for cellular processes [30,31].

PCBP1 plays a critical role in systemic iron homeostasis, as hepatocyte-specific PCBP1 knockout (KO) mice show low hepatic ferritin levels, mitochondrial dysfunction, and ROS-mediated lipid peroxidation [32,33]. The direct involvement of PCBP in intestinal ferritin regulation is not clear. However, PCBPs might indirectly regulate enterocyte iron levels by (a) delivering iron to PHDs that in turn modulate intestinal HIF-2α levels and (b) PCBP2 binding to both DMT1 and Fpn1 to promote iron cellular uptake [34]. More recently, it was shown that PCBP1 and 2 are essential genes for mouse development [35] and erythroid differentiation [36]. In addition, PCBP1 and nuclear receptor co-activator 4 (NCOA4) co-operatively regulate erythroid iron storage and erythropoiesis (discussed below). 

In addition to regulating intracellular iron trafficking, PCBPs are involved in post-transcriptional gene regulation, maintenance of mitochondrial stability, and are associated with a wide variety of pathophysiologies such as rheumatoid arthritis [37], amyotrophic lateral sclerosis (ALS) [38], and Huntington’s disease (HD), and certain neurodevelopmental disorders [39]. Although the iron chaperone and RNA-binding functions of PCBPs have been shown to be independently essential [40], it is not clear how these functions potentially contribute to the pathogenesis of the above-mentioned disorders.

## 3. Ferritinophagy—For Efficient Iron Release from the Store

Iron needs to be released from its storage form during conditions of scarcity or high demand. This involves proteolytic degradation of ferritin occurring primarily in the lysosome [41]. Earlier studies indicated that autophagic machinery are involved in ferritin degradation and subsequent iron release. More recently, NCOA4, originally known as nuclear receptor co-activator, was identified as a cargo receptor targeting ferritin for lysosomal degradation—a process coined as ferritinophagy [42]. NCOA4 colocalizes with ferritin in autophagosomes and lysosomes, and its in vitro or in vivo depletion inhibits ferritin delivery and degradation (Figure 1). The consequent reduction in ferritin turnover leads to a significant decrease in cytosolic LIP that ultimately alters cellular and systemic iron homeostasis [43,44]. NCOA4 expression and function are tightly regulated by intracellular iron status: (a) excess iron targets NCOA4 for ubiquitin-dependent turnover via its iron-dependent HERC2-mediated proteolysis [45]; (b) low iron increases NCOA4 mRNA levels via HIF-dependent transcriptional regulation [46,47].

NCOA4-mediated ferritin degradation is critical for erythroid differentiation and hemoglobinization in mice as these processes rely on efficient iron delivery and turnover in the RBCs [45,48]. In addition, both PCBP-mediated iron flux through ferritin and NCOA4-mediated ferritinophagic iron release occurs in a sequentially coordinated manner throughout the process of erythroid differentiation to maintain a consistent iron supply [48,49]. Whole-body NCOA4 KO mice develop microcytic hypochromic anemia, which is more severe in young mice compared to adults [47,50,51]. A recent study in erythroid-specific and tamoxifen-inducible systemic NCOA4 deletion mouse models reveals that NCOA4 maintains erythropoiesis by both cell-autonomous and non-autonomous mechanisms that explain how the compensatory mechanisms operate in age- or tissue-specific NCOA4 ablation [51].

NCOA4 plays a critical role in liver iron homeostasis in a HIF-dependent manner (both HIF1α- and HIF-2α), as evidenced by significant impairment in iron mobilization from ferritin in phlebotomized or iron-deficient hepatocyte-specific NCOA4 KO mice [46]. Additionally, our work shows that intestinal NCOA4 is exclusively induced by HIF-2α stabilizing conditions, such as dietary iron deficiency and chemically induced hemolytic anemia, and modulation of the intestinal NCOA4-HIF-2α axis could be therapeutically used for attenuation of systemic iron overload [47,52]. It is important to note here that although the role of NCOA4 in cellular and systemic iron mobilization is well studied in human cell lines and various mouse models, the role of NCOA4 in human iron disorders has not been well studied. In addition, although the significance of autophagy in cancer is well established and altered NCOA4 expression is reported in various cancer types, the role of ferritinophagy in tumorigenesis is yet to be clearly defined.

## 4. Ferroptosis—From the Perspectives of PCBP and NCOA4

Ferroptosis is an iron-dependent non-apoptotic form of cell death characterized by the accumulation of lipid peroxides [53]. Perturbations in cellular iron homeostasis via alteration of LIP levels and/or dysregulation in the network of iron-related proteins alter the cellular sensitivity to ferroptosis (Figure 1). Ferroptosis can be inhibited by limiting intracellular iron availability [54,55] and can be induced in vitro or in vivo by high iron [53,56]. As expected, cellular FTH expression profiles can be helpful in determining their sensitivity to ferroptosis and can be used for therapeutic targeting of ferroptotic pathways [57,58,59,60]. Studies show that induction of ferritinophagy promotes ferroptosis by increasing the levels of redox-active iron, which can be suppressed by NCOA4 ablation [61,62,63,64,65]. In contrast to hypoxic induction of ferritinophagy in the liver and the intestine [46,47], hypoxia increases ferritin expression in primary human macrophages via miRNA-mediated ferritinophagy inhibition and protects the cells from ferroptosis [66]. Several clinical studies suggest that high ferritin levels and altered ferroptosis gene expression are associated with poor prognosis of malignant and neurodegenerative disorders [67,68,69]. In addition, modulation of ferritinophagy to regulate cellular ferroptosis sensitivity offers novel avenues for cancer, cardiovascular and metabolic disorder management [70,71,72]. For example, itaconate, a well-known immunomodulator compound, has recently been identified as a novel inducer of ferritinophagy, which suppresses tumor growth in mice [73]. As iron chaperones, PCBPs provide redox-active iron to the downstream players and thus act as a positive regulator of ferroptosis [74]. Several studies also show that PCBPs limit ferritinophagy, and in vitro or in vivo depletion of PCBP induces ferroptosis [32,75,76]. From the translational standpoint, both iron chaperone and ferritinophagy functions are attractive therapeutic targets for disorders like cancer and neurodegeneration [77].

## 5. Mammalian Ferritin and the Gut Microbiome

The mammalian gastrointestinal tract is a host to trillions of microorganisms, including viruses, bacteria, fungi, and protozoa, collectively known as gut microbiota [78]. The microbes co-evolved with their mammalian hosts over thousands of years to develop a mutually commensal relationship and significantly contribute to hosting nutrient homeostasis and metabolism [79]. Gut microbiota has been shown to be directly involved in a wide variety of diseases, such as obesity [80], hypertension [81], inflammatory bowel diseases (IBDs) [82], and autism [83]. Through metabolic utilization of host-derived nutrients, gut microbiota produces a vast number of metabolites and small molecules, which interact with the host not only locally but also peripherally after their systemic absorption [84,85].

As regular and adequate iron supply through diet is critical for both, intense competition for the metal prevails between the host and microbes. Numerous studies have shown that iron availability influences the gut microbiome composition and function [86,87,88,89,90]. Human studies representing various geographic and pathologic backgrounds show that modulation of dietary iron levels influences links gut microbiome with the etiopathogenesis of a variety of disorders like dysbiosis, inflammation, and colorectal cancer [91,92,93,94]. For example, a recent human study involving nonalcoholic fatty liver disease (NAFLD) patients has shown that a positive association between serum ferritin levels and high liver fat accumulation is strongly connected with the gut microbiome: (a) host serum ferritin has both negative and positive associations with distinct bacterial families; (b) ferritin is connected to host-microbiota crosstalk on amino acid sensing pathways; (c) fecal microbiota transplantation (FMT) of human-derived bacteria in mice altered their iron metabolism gene signature [95].

**Figure 2:**
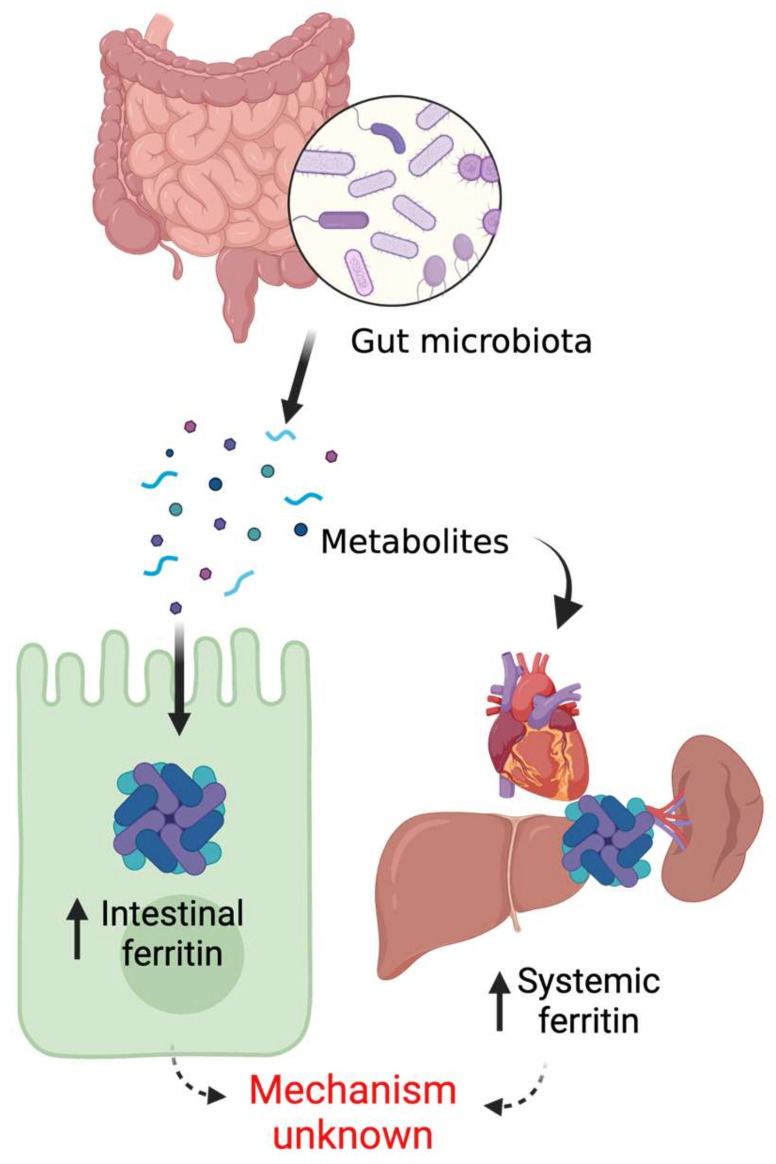
Gut microbial regulation of host ferritin.

Although the essentiality of iron for gut microbial survival in host intestinal lumen is well established, host-microbiota crosstalk on iron acquisition was only discovered recently. Deschemin et al. provided the first evidence that host intestinal iron sensing pathways are directly regulated by the commensals [96]. Our work demonstrated that gut microbial regulation of host systemic iron homeostasis takes place via microbial metabolite-mediated inhibition of intestinal HIF-2α signaling [97]. It is worth mentioning here that HIF-2α, an oxygen- and iron-dependent transcription factor, is the master regulator of intestinal iron absorption [98]. Strikingly, host intestinal as well as peripheral tissue ferritin expression is undetectable or significantly downregulated in germ-free or antibiotic-treated mice, which promptly returns to basal level following FMT from healthy donor mice [96,97]. These findings suggest that microbial presence in the gut is necessary for basal expression of host ferritin. Furthermore, recovery of ferritin levels in the microbiota-depleted mice by the systemic introduction of bacterial metabolites establishes the role of microbial metabolites in the regulation of host iron storage pathways [97] (Figure 2).

Ferritin is an important indicator of cellular redox balance, which is frequently altered in a wide spectrum of diseases, including chronic inflammation, metabolic disorders, and cancer [99,100]. Identification of specific microbial communities and bacteria-derived metabolites that modulate host ferritin would be potentially beneficial for pathophysiologic understanding as well as disease management. Successful development of in vitro co-culture with mammalian cells and in vivo colonization of germ-free gut models are needed to pinpoint the precise mechanisms of how gut microbiota or microbially derived metabolites regulate mammalian ferritin pathways.

## 6. Ferritin as a Novel Marker for COVID-19 Prognosis and Disease Severity

The ongoing global pandemic of coronavirus 2019 disease (COVID-19) caused by the acute respiratory syndrome coronavirus 2 (SARS-CoV-2) presents with heterogeneous clinical symptoms widely ranging from asymptomatic infection to fatal respiratory failure [101]. In addition to severe systemic inflammation caused by complex immunological changes, COVID-19 patients often present with dysregulated coagulation cascade system leading to further cardiovascular decompensation [102]. Massive systemic release of pro-inflammatory mediators such as interleukin (IL)-1β, IL-2, IL-6, IL-7, IL-10, tumor necrosis factor-α (TNFα), granulocyte colony-stimulating factor (G-CSF), etc., also known as cytokine storm [103], contributes to the acute respiratory distress syndrome (ARDS) [104]. In addition, many of these patients present with hyperferritinemia, which may result from hemolysis caused by SARS-CoV-2-induced inflammation [105,106]. Considering the combined presence of excessive cytokine release and hyperferritinemia on many occasions, it was proposed that COVID-19 should be considered as a spectrum of the hyperferritinemic syndromes [107,108,109].

Progressively high ferritin values, along with other inflammatory biomarkers, have been consistently reported to be associated with increased disease severity and mortality [110,111,112,113]. Recently, a scoring system for predicting the disease severity, namely, the Severe COVID Prediction Estimate (SCOPE) score, has been developed, which is composed of circulating levels of ferritin, C-reactive protein, D-dimers, and IL-6. SCOPE score values of 6 or more predict the progression to severe respiratory failure or death within 2 weeks of hospital admission for pneumonia [114]. Also, serum ferritin levels dropped in patients treated with tocilizumab, an IL-6 receptor antibody [115]. Interestingly, a study reports that patients with higher ferritin levels (than the median value of 1419 µg/L) benefitted most from tocilizumab treatment [116].

Altogether, these results establish ferritin assessment is a useful prognostic indicator that can be used as a screening test for the prediction of disease severity and mortality.

## 7. Conclusions and Perspectives

Ferritin plays essential roles in cellular and systemic iron homeostasis. As the iron storage protein and also as an acute phase reactant during inflammation, ferritin has been extensively studied. Over the last 15 years, several outstanding questions regarding ferritin–iron dynamics have been answered. Together, these mechanisms have allowed us to get fresh perspectives on iron-related disorders as well as infection and cancers. Sustained efforts to identify novel ferritin regulatory mechanisms will help us develop newer strategies for disease management.
